# Comparison of Limberg Flap and Karydakis Flap Repair in Pilonidal Sinus Surgery: A Prospective Case-Control Study

**DOI:** 10.7759/cureus.28933

**Published:** 2022-09-08

**Authors:** Ertugrul G Alkurt, Yaşar Murat Vardar, İshak Sefa Tüzün

**Affiliations:** 1 Department of General Surgery, University of Hitit Erol Olçok Training and Research Hospital, Çorum, TUR; 2 Department of General Surgery, University of Medical Sciences Haseki Training and Research Hospital, Istanbul, TUR

**Keywords:** pilonidal sinus surgery, karydakis flap, prospective study, pilonidal sinus treatment, limberg flap

## Abstract

Background

Pilonidal sinus disease (PSD) is a chronic inflammation and infection of the sacrococcygeal region. It often affects young adult males and produces clinical findings with abscess and discharge in the sacrococcygeal region or painful sinus tract in the natal cleft. The best surgical technique for sacrococcygeal PSD is still disputed. This study aimed to compare the Karydakis flap technique (KFT) and Limberg flap technique (LFT) used in the surgical treatment of the sacrococcygeal pilonidal sinus.

Methodology

A total of 140 patients diagnosed with pilonidal sinus between 2010 and 2012 were included in the study. The patients were divided into two groups, namely, LFT (n = 73) and KFT (n = 67). Preoperative findings of the patients, surgical findings, and short and long-term postoperative findings were recorded and statistically compared.

Results

Regarding cosmetic results, the Karydakis flap was better than the Limberg flap with a faster return to normal life. There was no statistical difference between the two groups concerning wound dehiscence, postoperative visual analog scale scores, seroma formation, and recurrence.

Conclusions

Considering the faster return to normal life and greater cosmetic satisfaction of the patients, the KFT should be chosen instead of the LFT as the standard technique in pilonidal sinus surgery.

## Introduction

Pilonidal sinus disease (PSD) is a chronic inflammation and infection of the sacrococcygeal region [1]. PSD was first described by Hodges in 1880 [2]. Although the actual incidence of PSD is not fully known, it has been reported to be 26 per 100,000 people in the United States [3]. Its incidence in Turkey is not known because of a lack of adequate investigation.

PSD occurs at least two times as frequently in men as in women, usually between the ages of 15 and 30 [4-7]. Although many theories have been proposed to explain the etiology of PSD, it has been now concluded that PSD has an acquired etiology [4].

The main goal of treatment is the early return of the patient to normal life and work, eliminating the recurrence of the disease. Several studies have aimed to determine how to best approach these treatment principles. As a result of these discussions, there are many different alternative treatments and surgical methods available for the treatment of the disease today [8,9]. Although many conservative and surgical methods have been described for the treatment of PSD, recurrence is still high and the search for an ideal treatment continues.

Both surgical and non-surgical methods are available for the treatment of PSD. Intracavitary injection of alcohol phenol and silver nitrate are non-surgical methods. Marsupialization, curettage after fistulotomy, primary closure after excision, and flap procedures are surgical methods. Successful treatment refers to the form of treatment wherein postoperative care is easy and cost-effective. It should be aimed at the patient to return to normal life in a short time [10].

This study aims to compare the common surgical treatment options for PSD, namely, the Karydakis flap technique (KFT) and the Limberg flap technique (LFT).

## Materials and methods

After obtaining approval from the Haseki Training and Research Hospital Clinical Research Ethics Committee (approval number: 168), a prospective case-control study was planned following the Declaration of Helsinki criteria. The study included patients who presented to the General Surgery Clinic of Haseki Education and Research Hospital between 2010 and 2012 and were diagnosed with chronic sacrococcygeal PSD.

Patients aged over 18, with no additional diseases, and with a body mass index (BMI) of 18-30 kg/m^2^ were included in the study. Recurrent cases, active infectious cases, and patients who could not be followed up after the operation were excluded from the study. A total of 140 pilonidal sinus patients were evaluated preoperatively and their records were maintained. Experienced surgeons were divided into two groups. The surgeons in the first group performed the repair with the Limberg flap, while the surgeons in the other group performed the repair with the Karydakis flap.

All patients were operated on under spinal anesthesia, the same suture materials were used during the operation, and a 12 f Redon drain was placed under the flap. The drains of all patients were removed on the first postoperative day. Stitches were removed on the 10th day in cases without complications.

Patients who were operated on for pilonidal sinus were monitored on the fifth day, tenth day, twentieth day, third month, sixth month, and twelfth month postoperatively. On the fifth, tenth, and twentieth days, the patients were called to the clinic and examined. Seroma, scar dehiscence, postoperative pain using the visual analog scale (VAS) score, and purulent discharge at the incision site were recorded. Recurrence control was performed in patients who came to the clinic for control.

Pain at the operation site and cosmetic dissatisfaction were assessed using a VAS score from 1 to 10. The degree of cosmetic satisfaction was recorded. For cosmetic satisfaction, we asked the patients, “Please describe your satisfaction with the operation scar as compared to pilonidal sinus disease.” The feeling of numbness was similarly scored from 1 to 10 points, as well as the level of satisfaction. Patients who did not come to the outpatient clinic were surveyed by telephone. Patients with suspected recurrence were called to the clinic. Patients who did not come to the outpatient clinic and could not be reached by phone were excluded from the study.

Preoperative preparation

All patients included in the study were informed in detail about the study before the operation and their written consent was obtained. Hair was cleaned by shaving the patients in the evening before the operation or on the morning of the operation. Cefazolin sodium IM (1 g) was administered prophylactically 30 minutes before the operation. All surgeries were performed under spinal anesthesia. The operation area was cleaned by wiping it at least two times with the help of gauze moistened with polyvinyl iodine. Before starting the operation, the incision borders were drawn with a sterile pen, including the sinus.

Surgical technique

Limberg Flap Technique (Group 1)

**Figure 1:**
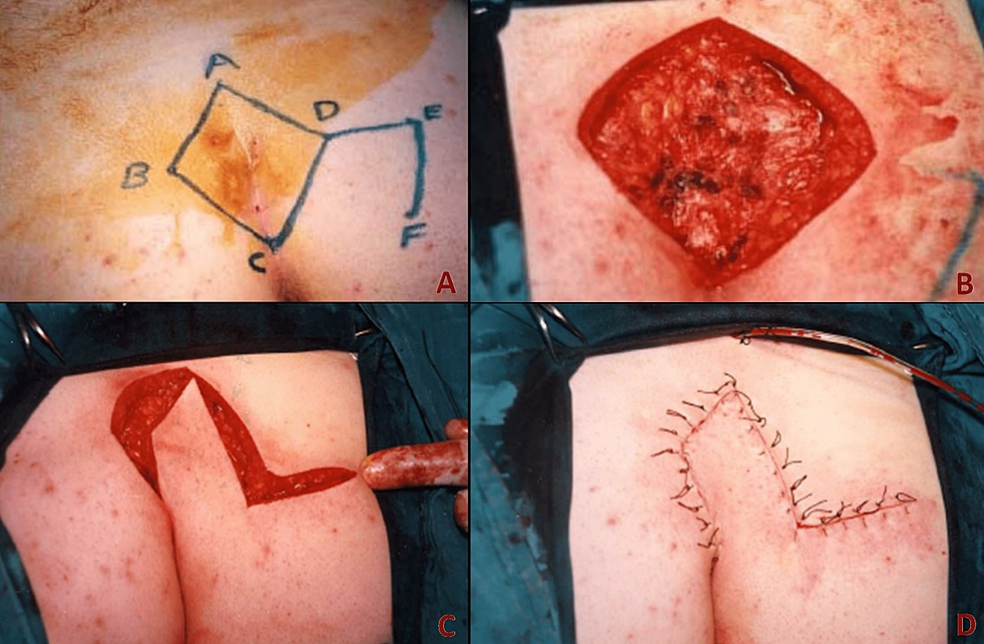
Limberg flap technique. A: Marking equilateral triangles before the excision. B: Excision of the sinus along with the rhomboid. C: Creating a rotational flap. D: Appearance of the wound at the end of the operation.

The sinus tract was determined by injecting methylene blue into the sinus with the aid of a syringe. A rhombohedral skin incision was made with a sinus opening in the center. Guided by this incision, all tissues were removed by descending into the presacral fascia. Bleeding was cauterized. The horizontal bisector of the ABCD parallelogram shown in Figure 1A was extended by an incision made on the right gluteus to the length of the AB side to reach point E, as shown in Figure 1A. This point is parallel to the CD edge and has the same length. The incisions were deepened to include the gluteal muscle fascia and the fascia was released. Thus, the rhomboid flap was prepared. The wound was irrigated with copious amounts of saline. Patches holding the gluteus in traction were opened to facilitate closure. Flap transposition was done so that the FD points came together. After placing a vacuum Redon drain under the flap, a separate hole was opened and taken out. It was closed by suturing with 2/0 vicryl to bring the fascia and subcutaneous tissues together. 3/0 polypropylene material was used in skin suturing. The skin was washed with physiological saline and dried, and after wiping it with povidone-iodide again, it was covered with sterile gauze (Figure 1).

Karydakis Flap Technique (Group 2)

**Figure 2:**
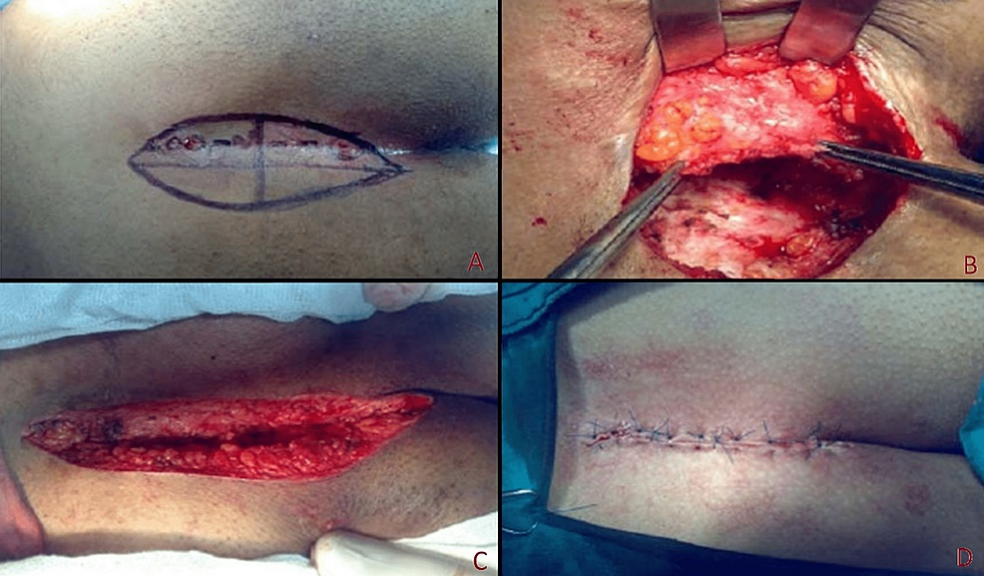
Karidakis flap technique. A: Simple elliptical excision. B: Flap elevation. C: Approximation and closure of the wound. D: Final view of the surgical area.

To ensure complete resection of the pilonidal sinus, methylene blue was injected into the fistula before the operation and the lesion was stained. An asymmetric and biconcave incision was made, as described by Karydakis [10]. If there was a secondary opening or induration on one side of the ellipse, the incision was shifted in that direction. The excised lateral edge of the ellipse was tried to be symmetrical with the medial edge, and, for this, more skin and adipose tissue were excised around the sinus when necessary. Thus, it was ensured that the suture line was vertical. The lateral margin was excised symmetrically with the medial margin. Next, a 1 cm deep and a 2 cm inward flap of the medial edge of the wound was prepared using cautery to extend across the entire incision. Absorbable sutures (2/0 polyglycolic acid) were used to cover both surfaces from the adipose tissue in the prepared flap. Subsequently, a series of sutures were placed along the entire flap and tied, passing through the midline of the presacral fascia. An assistant pushed the flap from its base to the presacral fascia, allowing the sutures to be easily tied. A vacuum Redon drain was placed on this suture line and removed from the lower end of the wound. The second row of sutures (2/0 polyglycolic acid) was placed between these two layers to approximate the lower surface of the flap and the lateral adipose tissue. A useful modification here is to pass the sutures through the deep fascia when placing the second row of sutures. Thus, the flap was flattened and the dead space was eliminated. When this second suture line was tied, the drain was completely covered. Vertical U-shaped sutures were placed on the skin with intermittent 3/0 prolene. Care was taken not to leave any gaps between the sutures in the skin sutures (Figure 2). The skin was washed and dried with saline, and after wiping it with povidone-iodide again, it was covered with sterile gauze.

Statistical analysis

Data analysis was performed using SPSS for Windows 22 (IBM Corp., Armonk, NY, USA). The Kolmogorov-Smirnov test was performed to test whether the data were normally distributed. Categorical variables were presented with frequency distribution (numbers and percentages), and descriptive statistics were used for numerical variables. The Mann-Whitney U test was used to assess differences in numerical variables between the groups, and the Chi-square test and the Student’s t-test were used to compare categorical variables. A p-value of <0.05 was considered significant.

## Results

After applying the inclusion and exclusion criteria, 123 (87.9%) of the 140 patients who made up the study group were male and 17 (12.1%) were female. There were 67 (91.8%) males and six (8.2%) females in Group 1 (n = 73), 56 (83.6%) male, and 11 (16.4%) females in Group 2 (n = 67).While the mean age of Group 1 was 25.55 ± 6.78 years, the mean age of Group 2 was 26.4 ± 8.48 years. No statistical difference was found between the gender distribution and age between the groups (p = 0.138 and p = 0.514, respectively).

The mean BMI was 24.6 ± 3.74 kg/m^2^ in Group 1 and 24.2 ± 3.54 kg/m^2^ in Group 2. There was no statistical difference between the groups (p = 0.574).

No statistical difference was detected between groups regarding the number of sinuses (p = 0.936). Group 1 had one sinus pit in 31 patients, and two and more sinus pits in 42 patients. Group 2 had one sinus pit in 28 patients, and two or more sinus pits in 39 patients.

Wound dehiscence was observed in five patients in Group 1 and seroma in eight patients on the fifth day, while wound dehiscence was observed in two patients and seroma in five patients in Group 2. There was no statistically significant difference between the groups regarding wound dehiscence and seroma formation (p = 0.295 and p = 0.476, respectively). In addition, no statistical difference was detected between the postoperative VAS scores (Table 1).

**Table 1:** Comparison of Limberg flap and Karydakis flap techniques. *Student’s t-test; **Chi-square test; ***Mann-Whitney U test. BMI: body mass index; VAS: visual analog scale

		Group 1 (n = 73)	Group 2 (n = 67)	P-value
Female		6 (8.2%)	11 (16.4%)	0.138*
Male		67 (91.8%)	56 (83.6%)	
Age, years		25.5 ± 6.78	26.4 ± 8.48	0.514*
BMI, kg/m^2^		24.61 ± 3.74	24.26 ± 3.54	0.574**
Sinuses, n	1	34 (42.5%)	28 (41.8%)	0.936**
	2 or more	42 (57.5%)	39 (58.2%)	
Operation time	0–60 minute	47 (64.4%)	51 (76.1%)	0.13**
	60–90 minute	26 (35.6)	16 (23.9%)	
Wound dehiscence	(-)	68 (93.2%)	65 (97%)	0.295***
	(+)	5 (6.8%)	2 (3%)	
Seroma	(-)	65 (89%)	62 (92.5%)	0.476***
	(+)	8 (11%)	5 (7.5%)	
Postoperative VAS		1.15 ± 0.6	1 ± 0.65	0.17**
Cosmetic result	Not satisfied	16 (21.9%)	3 (4.5%)	0.003**
	Satisfied	57 (78.1%)	64 (95.5%)	
Recurrence	(-)	68 (93.2%)	61 (91%)	0.644**
	(+)	5 (6.8%)	6 (9%)	
Back to work day		11.64 ± 1.91	10.56 ± 1.19	0.001**

As a result of the evaluations made in the third-month, sixth-month, and first-year follow-ups of the groups, there was a statistical difference between the two groups concerning cosmetic satisfaction. Patients in Group 2 were more satisfied with their cosmetic results (p = 0.003).

One year after the operation, recurrence was observed in five patients in Group 1 and six patients in Group 2 during the controls performed in the outpatient clinic. In our study, no significant difference was found between the groups regarding recurrence (p = 0.644).

The mean return to normal life and work was 11.61 ± 0.91 days in Group 1 and 10 ± 1.19 days in Group 2 in patients who were operated on with the LFT and KFT. In our study, it was found that starting work was significantly earlier in Group 2 than in Group 1 (p = 0.001).

## Discussion

In this prospective case-control study, there was no statistical difference between the two groups concerning wound dehiscence, seroma formation, and recurrence. Regarding cosmetic results, the Karydakis flap was better than the Limberg flap, and the return to normal life was faster.

Although many conservative and surgical methods have been described in the treatment of pilonidal sinus, the recurrence rate is still high, and the search for an ideal treatment continues [11-13]. In the treatment of chronic PSD with conservative methods, because the wound healing is completed in an average of 40-50 days, return to work is delayed. Cure rates have been reported to be approximately 70% [14]. Blumberg [15] in his study with 11 patients reported that seven patients treated with conservative methods resulted in recurrence. The patients were followed up between seven weeks and six years, and recovery was achieved between five and 16 weeks. The recurrence rates in the studies were high. Therefore, the general approach for an effective treatment method is excision of the pilonidal cyst and closure of the formed cavity with flap methods. In these methods, hematoma, temporary wound dehiscence, and wound infection are important problems in the postoperative period.

Karydakis [16] reported that this method is an easy technique, the suture line remains lateral, it has the advantages of early recovery and early return to work, and the recurrence rate is as low as 1%. In studies that reported the recurrence rate as 5%, this high rate was attributed to the poor application of the technique and the shift of the suture line to the middle [16,17]. Many surgeons have performed Karydakis flap surgery. Kitchen [17] performed Karydakis flap surgery on 141 patients and followed up for five months to six years. As early postoperative complications, he reported hematoma in seven (5%) patients and wound infection in six (4.5%) patients. Regarding late complications, wound numbness was found in 17 (12%) patients, slow healing in four (3%) patients, and recurrence in five (4%) patients.

In a study of 27 patients operated by Anyanwu et al. [18] using the KFT, no postoperative recurrence or complication was reported in any of the patients. LFT was first used by Azab in 1984. Azab et al. [19] applied Limberg flaps to patients and found no recurrence in follow-ups ranging from six months to three years. In this study, minor wound infection was found in five (17%) cases and major wound infection was found in one (3%) case as complications. In the study by Lieto et al. [20], which included 310 patients, the authors showed that the hospital stay was short, the postoperative pain was low, and the recurrence was low The average length of stay in the hospital was reported to be 10 days. In the treatment of pilonidal sinus with the LFT, various authors have reported wound complications as 0-17%, recurrence rates as 0-5%, and average hospitalization times of five to seven days [20-23].

There are also disadvantages of the LFT. In a study by Erdem et al. [23] involving 63 patients, it was reported that 63% of the patients were not satisfied with the cosmetic results. In our study, the LFT and the KFT were compared. While recurrence was observed in five patients in the LFT group, recurrence was observed in six patients in the KFT group, and no statistically significant difference was found between the groups in terms of recurrence. In the early follow-up of patients who were operated on with the KFT and LFT, seroma developed in eight patients who were operated on with the LFT, while seroma developed in five patients who were operated on with the KFT.

PSD is one of the most frequently encountered and operated diseases in surgical clinics. Although the treatment of this disease shows many variations, the preference for flap methods is increasing. Our study has shown that KFT, which can be easily applied in the surgical treatment of PSD, has a low complication rate, has high cosmetic satisfaction, enables the early return to normal life and work, and can be safely applied in the treatment of this disease.

There are some limitations of our study. First, long-term results should be evaluated to fully evaluate recurrence. We did not use an objective criterion for cosmetic evaluation other than the VAS score. The assessment of numbness was not objective. However, the study has the advantages of being a prospective study and the number of patients.

## Conclusions

We found that the KFT is advantageous over the LFT in terms of return to work. Therefore, it was determined that the KFT is an effective and advantageous surgical procedure in the treatment of pilonidal sinus.
